# Response Surface Optimization of Fly Ash–Carbide Slag–Bentonite-Based Whole-Tailings Backfill: Multi-Objective Mix Design and Microstructural Mechanism

**DOI:** 10.3390/ma19153346

**Published:** 2026-08-06

**Authors:** Junhui Yao, Jianyuan Jin, Yin Chen, Zulyar Ilxat, Hui Chen

**Affiliations:** 1 Key Laboratory of Green and Efficient Mining and Ecological Restoration in High-Altitude Arid Regions of Xinjiang, Urumqi 830047, China; rockman@xju.edu.cn (J.Y.); jinjianyuan2001@163.com (J.J.); zulyar233@163.com (Z.I.); 2 School of Geology and Mining Engineering, Xinjiang University, Urumqi 830047, China; 3 Xinjiang Kalatongke Mining Co., Ltd., Altai 836100, China; xjyscy@163.com

**Keywords:** fly ash, carbide slag, bentonite, response surface methodology, multi-objective optimization, microstructural mechanism

## Abstract

Reducing cement consumption in mine backfill materials and improving the synergistic utilization of multi-source solid wastes are important for developing low-carbon backfill systems. In this study, fly ash–carbide slag–bentonite-based whole-tailings backfill (FCB) was optimized using a Box–Behnken response surface design. The effects of replacement ratio, FA/CS ratio, and bentonite content on fluidity, setting time, and 28 d uniaxial compressive strength were investigated, and the microstructural mechanism was analyzed by XRD and SEM. The results show that all quadratic models are statistically significant, with the strength model exhibiting the best fitting and predictive performance. The replacement ratio and bentonite content are the main factors affecting FCB performance, while the FA/CS ratio plays a secondary role. Strength decreases with increasing replacement ratio, whereas bentonite content shows a quadratic effect on fluidity and strength. Multi-objective optimization gives an optimal mix of A = 0.58, B = 2.71, and C = 4.99%, with validation errors below 5%. XRD and SEM results indicate that C-(A)-S-H gel and AFt are the main hydration products. Lower replacement ratios and appropriate bentonite contents were associated with a more continuous matrix morphology in the selected SEM regions and higher compressive strength.

## 1. Introduction

With the increasing intensity of mineral resource exploitation, goaf stability control and tailings disposal have become critical issues in green mining. Cemented backfill technology can simultaneously provide goaf support, reduce tailings disposal, and control environmental risks. It has therefore become an important technical approach for green mining in modern mines [1,2]. Compared with conventional tailings storage, backfill mining can effectively relieve the operational pressure on tailings ponds and improve mining safety and resource recovery efficiency [3]. Among different backfill methods, whole-tailings backfill eliminates the tailings classification process and offers advantages such as a simplified process, high tailings utilization, and strong engineering adaptability. As a result, it has attracted increasing attention in metal mine backfilling [4].

Ordinary Portland cement remains the most commonly used binder in mine backfill. However, its high cost and carbon emissions have restricted the wider application of backfill technology [5,6]. Previous studies have indicated that binders usually account for a major proportion of backfill cost, and that reducing cement consumption and developing alternative cementitious systems are important approaches for low-cost backfill [5]. Meanwhile, the cement industry remains an important source of industrial CO_2_ emissions. The development of low-carbon cementitious materials has therefore become a common demand in both construction materials and mine backfill fields [6,7]. In recent years, industrial solid-waste-based alkali-activated materials and low-carbon composite cementitious systems have received increasing attention because of their potential for resource utilization, emission reduction, and favorable mechanical performance. These materials have also shown promising application prospects in mine backfill [8].

Among various industrial solid wastes and functional mineral materials, fly ash (FA), carbide slag (CS), and bentonite (BE) have distinct material characteristics, which provide a basis for developing low-carbon backfill binders. Sun et al. prepared FA-based geopolymer paste backfill materials and showed that FA can act as an important aluminosilicate precursor in cementitious reactions, forming a relatively complete microstructural bonding framework [9]. Liu et al. investigated the early strength formation and hydration mechanism of alkali-activated slag/FA backfill materials and reported that the release of FA activity and gel formation played important roles in strength development [10]. For high-calcium activating components, Zeng et al. studied an FA-CS low-carbon cementitious system through pre-depolymerization and pre-dissolution methods and found that CS could promote FA reaction and improve material performance [11]. Li et al. further indicated that CS-activated GGBS-FA systems exhibited favorable strength development, microstructure, and sustainability [12]. In addition, Hasan et al. reviewed the application of BE in cement-based composites and suggested that BE can improve material performance through filling effects, water retention, and rheological regulation [13]. Fode et al. also showed that both raw and calcined BE can affect the mechanical and durability properties of cementitious systems [14]. Therefore, FA can provide a potentially reactive aluminosilicate source, CS can provide calcium and an alkaline environment, and BE can contribute to rheological regulation and structural refinement. These characteristics indicate a certain functional complementarity among the three components.

In addition to material composition, the performance of backfill is affected by the combined action of multiple factors. Variations in the proportions of different solid-waste components can alter not only slurry flowability and setting behavior, but also hydration product formation, pore structure, and strength development in the hardened matrix. Therefore, an appropriate mix design method is necessary to identify the main factors and their interactions in the development of low-carbon backfill materials. Response surface methodology (RSM) can establish relationships between factors and responses with relatively few experiments, while also evaluating main effects, quadratic effects, and interaction effects. It has therefore been increasingly applied to mix proportion optimization of backfill materials [15]. Huang et al. used RSM-BBD for multi-objective optimization of cemented neutralization slag backfill and demonstrated that this method can effectively evaluate factor interactions and determine optimal mix parameters [16]. Sun and Fall applied RSM to characterize and optimize slag-based fiber-reinforced cemented tailings backfill, confirming the suitability of RSM for the coordinated optimization of workability and strength [17]. Zhao et al. applied RSM to optimize an anhydrous phosphogypsum-based backfill incorporating carbide slag and blast furnace slag, demonstrating the applicability of this method to multi-component backfill systems [18]. These studies indicate that RSM can provide effective methodological support for the mix design and performance optimization of multi-source solid-waste backfill materials.

Although substantial progress has been made in the development of solid-waste-based binders for mine backfill, previous studies have mainly focused on fly ash-based geopolymers, alkali-activated slag–fly ash binders, carbide slag-activated slag systems, or other single- and binary-component formulations. In comparison, the combined incorporation of fly ash, carbide slag, and bentonite into cemented whole-tailings backfill has received relatively limited attention. In particular, the coupled influences of the cement replacement ratio, FA/CS ratio, and bentonite content on slurry fluidity, setting behavior, and compressive strength have not been systematically quantified. The relationships between these compositional variables, the resulting hydration products, and the microstructural characteristics of the hardened backfill also remain insufficiently clarified.

Accordingly, the aim of this study is to develop and optimize a fly ash–carbide slag–bentonite-modified whole-tailings backfill with reduced cement consumption while maintaining acceptable workability, setting behavior, and mechanical performance. To achieve this aim, the specific objectives are: (1) to quantify the linear, interaction, and quadratic effects of the cement replacement ratio, FA/CS ratio, and bentonite content on fluidity, initial setting time, final setting time, and 28 d uniaxial compressive strength; (2) to establish response surface models and determine a balanced mix proportion through multi-objective optimization; and (3) to relate the observed macroscopic performance variations to the phase composition and microstructural characteristics identified by XRD and SEM.

The scientific novelty of this study lies in the systematic investigation of the ternary FA–CS–BE system by combining mix proportion optimization, engineering performance evaluation, and microstructural analysis. Unlike previous studies that predominantly considered alkali-activated fly ash/slag systems or binary solid-waste binders, the present study evaluates a system combining an aluminosilicate-rich component, a calcium-rich alkaline component, and a fine mineral regulator. It further integrates the multi-response statistical optimization of fresh and hardened properties with microstructural interpretation. This approach provides additional insight into how the three compositional variables collectively influence the engineering performance and structural development of cemented whole-tailings backfill.

## 2. Materials and Methods

### 2.1. Raw Materials

The raw materials used in this study included composite Portland cement (CE), Class I fly ash (FA), carbide slag (CS), calcium bentonite (BE), and whole tailings (TA) obtained from a copper mine. CE, FA, CS, and BE were used as the binder components, while TA served as the backfill aggregate. Prior to testing, all powder materials were oven-dried, and the tailings were also dried to minimize the influence of moisture fluctuation on the test results.

The major chemical compositions of the raw materials were determined by X-ray fluorescence spectroscopy (XRF) using a Rigaku ZSX Primus III + spectrometer (Rigaku Electric Co., Ltd., Tokyo, Japan) with the pressed-pellet method. The results were reported in the conventional oxide form and were not normalized on a calcined basis because loss on ignition was not measured. Therefore, the oxide notation in Table 1 represents the conventional reporting format of XRF analysis rather than direct confirmation of the oxidation states of the corresponding elements. The main chemical compositions of the raw materials are presented in Table 1. CE contained a relatively high CaO content, indicating its basic cementitious capacity. FA was mainly composed of SiO_2_ and Al_2_O_3_, exhibiting the typical characteristics of an aluminosilicate solid waste with potential pozzolanic activity [19,20]. CS contained 93.08 wt.% CaO, showing a pronounced calcium-rich composition and acting as an important calcium source in the system [21,22]. BE mainly consisted of SiO_2_, Al_2_O_3_, and a certain amount of CaO, reflecting the compositional characteristics of calcium-based clay minerals [23]. TA was dominated by SiO_2_ and also contained certain amounts of Al_2_O_3_ and Fe_2_O_3_, showing the typical characteristics of siliceous tailings.

**Figure 1:**
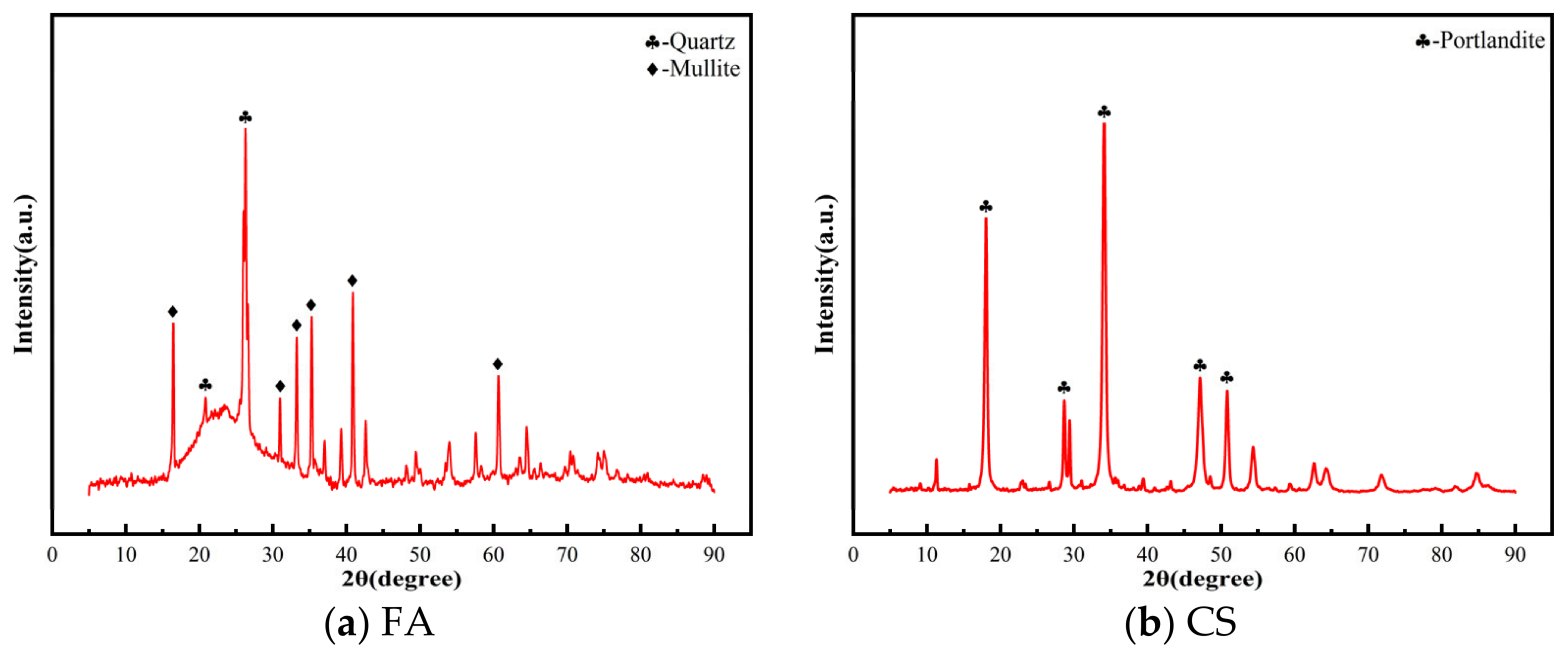

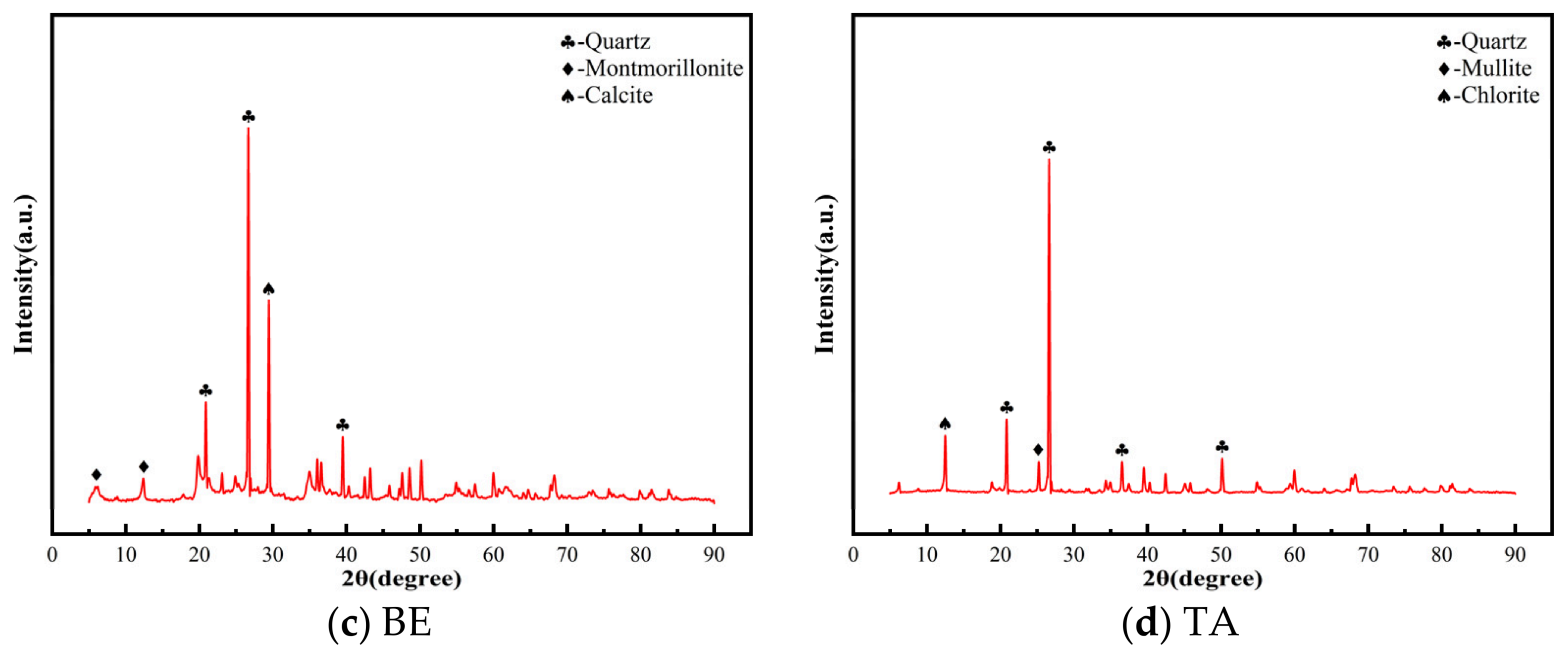
XRD patterns and identified major crystalline phases of the raw materials.

The XRD patterns of the raw materials are shown in Figure 1. FA exhibited a broad diffuse hump within the range of approximately 20–35° 2θ, indicating the presence of an amorphous aluminosilicate glassy phase with potential pozzolanic reactivity. The crystalline reflections superimposed on this diffuse background were mainly assigned to quartz and mullite [20]. In particular, the reflections near 20.8° and 26.6° 2θ were mainly associated with quartz, while several other reflections within and beyond this region were attributed to mullite. The XRD pattern of CS was dominated by the characteristic reflections of portlandite. The main reflections located at approximately 18.0°, 28.7°, 34.1°, 47.1°, and 50.8° 2θ indicate that calcium was predominantly present in the crystalline form of Ca(OH)_2_. Therefore, CS can provide both a calcium source and an alkaline environment for the cementitious reactions [21,22]. BE exhibited characteristic reflections of montmorillonite, together with prominent quartz and calcite peaks. The low-angle reflections were mainly associated with the layered structure of montmorillonite, whereas the reflections near 20.8° and 26.6° 2θ were assigned to quartz. The reflection near 29.4° 2θ was attributed to calcite. These mineralogical characteristics are consistent with those of calcium bentonite [23]. TA was mainly composed of quartz, accompanied by minor mullite and chlorite. The dominant reflections near 20.8° and 26.6° 2θ were assigned to quartz, while the weaker reflections were associated with mullite and chlorite. The high proportion of quartz and other crystalline silicate minerals indicates that TA primarily acts as a skeletal aggregate and filling component in the backfill system.

**Figure 2:**
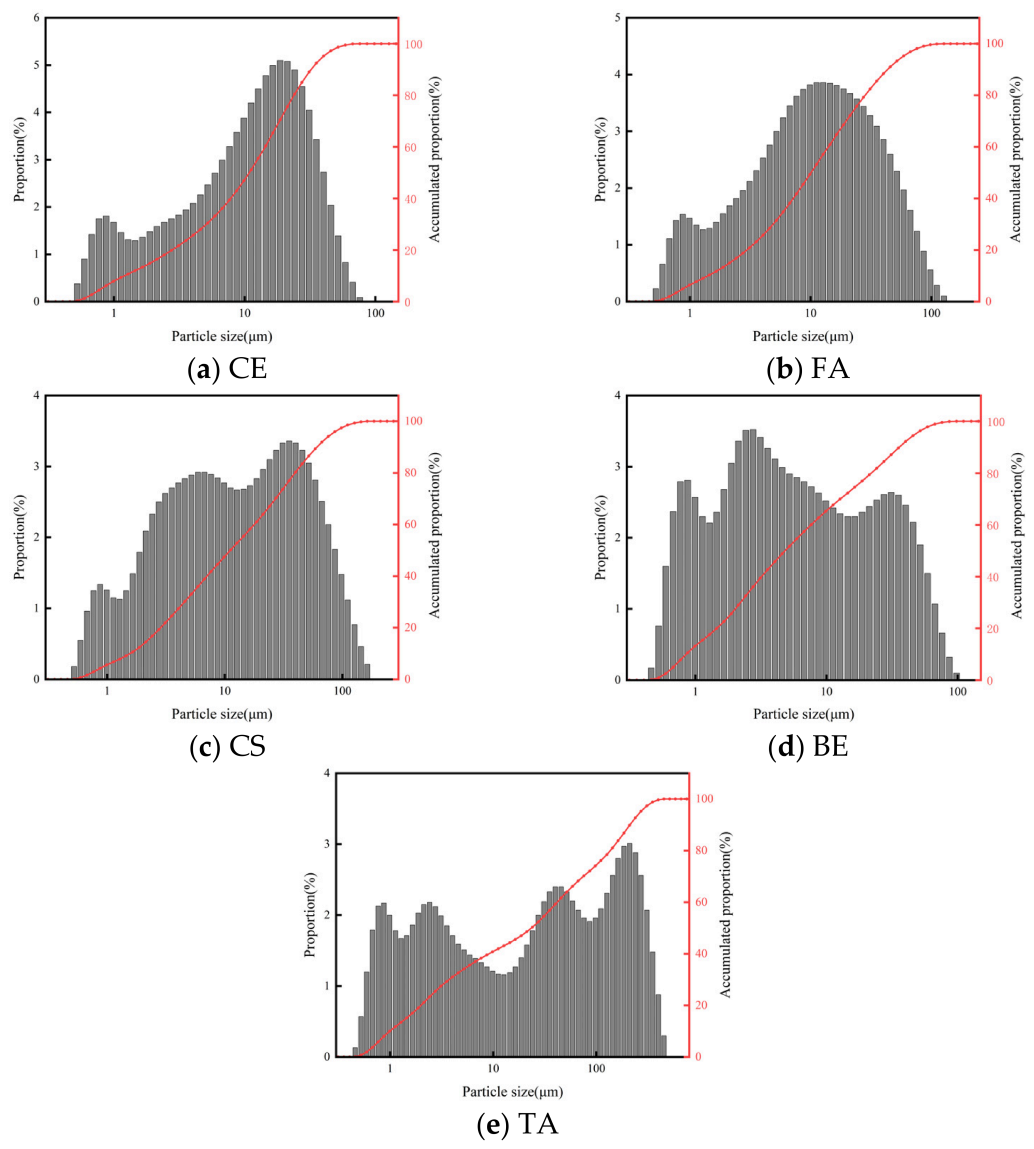
Particle size distributions of the raw materials.

The particle size distributions of the raw materials are presented in Figure 2. CE, FA, CS, and BE were mainly composed of fine particles. BE exhibited a relatively fine and concentrated particle size distribution, whereas TA showed a wider particle size distribution containing both fine and coarse particles. These differences may influence particle arrangement and slurry behavior. However, particle size distribution alone cannot be used to determine the specific surface area, adsorption capacity, or quantitative packing density of the raw materials.

Based on the above chemical compositions, phase characteristics, and particle size distributions, FA mainly acts as an aluminosilicate active component participating in potential cementitious reactions. CS provides a high-calcium environment and alkaline activation conditions. BE influences the slurry state and hardened structure through fine particle filling, water retention, and rheological regulation. TA mainly serves as the skeletal support and pore-filling component. The different constituents exhibit complementary roles in both chemical reactions and physical filling, providing a material basis for constructing a low-carbon FCB backfill system.

All raw materials were oven-dried before batching; however, their residual moisture content, water absorption, particle density, and BET specific surface area were not independently measured. Therefore, the related interpretations of water demand, adsorption, and particle-packing effects should be regarded as qualitative and represent a limitation of the present study.

### 2.2. Response Surface Experimental Design

Response surface methodology (RSM) was employed to investigate the effects of multiple compositional variables on the workability and mechanical performance of fly ash–carbide slag–bentonite-based whole-tailings backfill (FCB). RSM enables quantitative relationships between experimental factors and response variables to be established with a limited number of experimental runs and allows the linear, interaction, and quadratic effects of the factors to be evaluated [15].

Based on preliminary tests and engineering requirements, the binder-to-tailings mass ratio was fixed at 1:5, the slurry mass concentration was maintained at 82%, and the superplasticizer dosage was fixed at 0.2% of the total mass of solid materials. Under these conditions, the replacement ratio (A), FA/CS ratio (B), and bentonite content (C) were selected as the experimental factors. Specifically, A represents the replacement level of cement by the combined amount of FA and CS, B denotes the mass ratio of FA to CS, and C represents the mass percentage of BE in the total solid materials.

More specifically, factor A was calculated as the combined dry mass of FA and CS relative to the dry mass of cement, factor B was the dry mass ratio of FA to CS, and factor C was the mass percentage of BE in the total dry solids. The binder was defined as the combined mass of CE, FA, CS, and BE.

To illustrate the absolute mixture composition, the optimized mixture with A = 0.58, B = 2.71, and C = 4.99% can be expressed by taking the cement content as 1.000 mass part. The corresponding amounts of FA, CS, BE, whole tailings, mixing water, and superplasticizer were 0.424, 0.156, 0.675, 11.276, 2.970, and 0.027 mass parts, respectively. The total binder content was therefore 2.255 mass parts, and the whole-tailings content was five times the binder content. The other experimental mixtures can be reproduced in the same manner using the A, B, and C values listed in Table 2 and Table 3.

At a slurry mass concentration of 82%, the nominal total water-to-binder ratio was 1.317 for all mixtures. Because all powder materials and tailings were oven-dried before mixing, no correction for initial moisture content was applied. However, an absorption-corrected effective free-water-to-binder ratio could not be determined because the material-specific water-absorption data were unavailable.

A Box–Behnken design (BBD) was selected because the present study involved three quantitative factors at three levels and required the estimation of linear, interaction, and quadratic effects. For a three-factor system, BBD provides sufficient information for fitting a second-order polynomial model while requiring a relatively small number of experimental runs [24,25]. The design consisted of 17 experimental runs, including five replicated center points for estimating pure error and evaluating experimental repeatability and model stability.

Another advantage of BBD is that it does not include experimental combinations in which all three factors are simultaneously set at their extreme levels. This feature was considered appropriate for the present system because preliminary tests indicated that extreme factor combinations could result in mixtures with unsuitable fluidity, prolonged setting, or markedly reduced strength. In comparison, a conventional rotatable central composite design may introduce axial points outside the selected practical factor ranges, whereas a face-centered central composite design includes corner points at which all three factors simultaneously reach their extreme levels. Therefore, BBD was selected by considering the requirements of quadratic-model estimation, experimental efficiency, and the practical feasibility of the tested mixtures.

Each factor was assigned three coded levels of −1, 0, and 1. The corresponding actual values are presented in Table 2. Factor A was set at 0.5, 1.0, and 1.5; Factor B was set at 2, 3, and 4; and Factor C was set at 2%, 4%, and 6%, respectively.

Fluidity, initial setting time, final setting time, and 28 d uniaxial compressive strength were selected as the response variables. Based on the experimental results, second-order polynomial regression models were established to describe the relationships between the experimental factors and each response. Analysis of variance (ANOVA) was used to evaluate the statistical significance, adequacy, and lack of fit of the developed models. Response surface and contour plots were subsequently generated to illustrate the individual and interactive effects of the factors. Finally, numerical multi-objective optimization was performed to determine a mix proportion that balanced slurry workability, setting behavior, and mechanical performance [15,25].

### 2.3. Specimen Preparation and Test Methods

**Figure 3:**
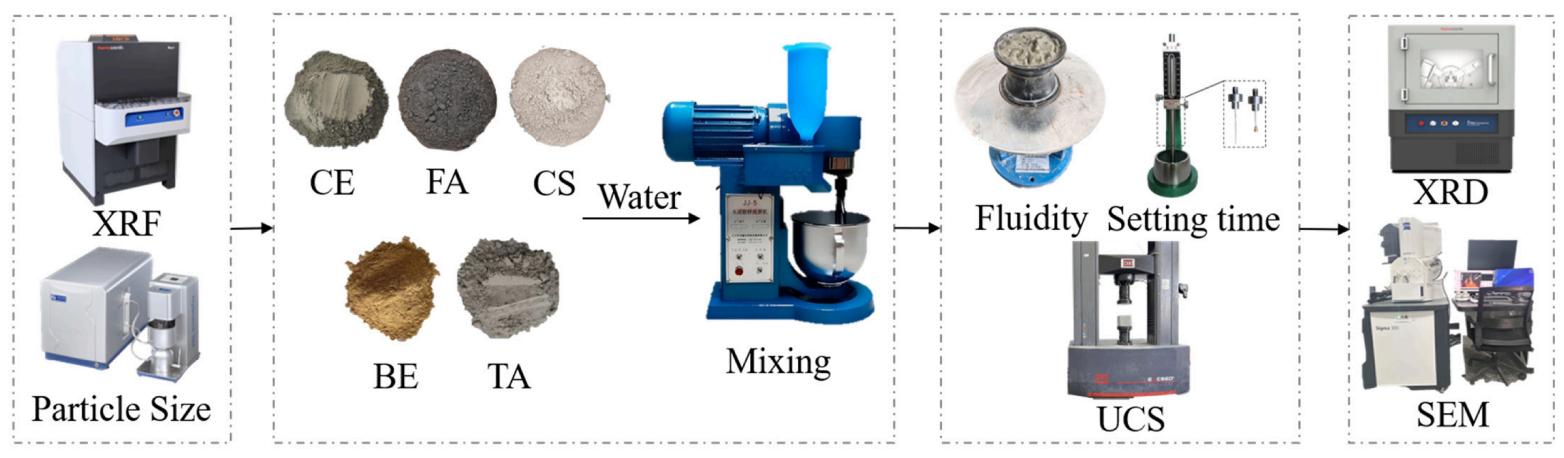
Preparation and testing procedure of FCB specimens.

The preparation and testing procedures of FCB specimens are illustrated in Figure 3. For each mix, CE, FA, CS, BE, and TA were weighed according to the designed proportions. The dry materials were first mixed thoroughly, followed by the addition of mixing water and superplasticizer. The mixture was then stirred using a planetary mixer until a uniform and stable slurry was obtained. To ensure comparability among different mixtures, the slurry mass concentration was maintained at 82%, and the superplasticizer dosage was fixed at 0.2% of the total mass of solid materials throughout the experiments.

After mixing, the slurry was used for fluidity testing, setting time measurement, and specimen casting. Fluidity was measured in accordance with GB/T 2419-2005 [26]. Setting time was determined using a Vicat apparatus following GB/T 1346-2011 [27], including both initial and final setting times. Uniaxial compressive strength tests were conducted in accordance with GB/T 50081-2019 [28].

Cylindrical specimens with dimensions of Φ50 mm × 100 mm were prepared and cured to the specified age. The compressive tests were performed using an MTS E45.605 testing machine(MTS Systems Corporation, Eden Prairie, MN, USA) and the compressive strength was calculated based on the peak load. For each mix, three parallel specimens were tested, and the average value was reported. To reduce experimental errors, the specimen preparation, curing, and testing procedures were carried out under consistent conditions. When significant dispersion was observed among parallel specimens, the specimen preparation quality and testing process were rechecked to ensure data reliability.

Considering the objectives of this study, the 28 d uniaxial compressive strength was used as the primary mechanical response in the response surface optimization and subsequent analysis. To investigate the microstructural characteristics of FCB, representative specimens at 28 d were selected for XRD and SEM analyses. XRD was conducted using a Rigaku Ultima IV diffractometer with Cu-Kα radiation (λ = 1.54060 Å) to identify phase composition and hydration products. The XRD patterns were processed using Jade software (version 9.0.2; Materials Data, Inc., Livermore, CA, USA). The major crystalline phases were tentatively identified by comparing their characteristic peak positions with reference diffraction patterns. For regions containing overlapping reflections, particularly within the range of 20–35° 2θ, phase assignments were based on the correspondence of multiple characteristic peaks rather than on a single reflection. SEM observations were performed using a ZEISS Sigma 300 field-emission scanning electron microscope (ZEISS, Oberkochen, Germany) to qualitatively compare the morphology of the reaction products, visible voids, particle contacts, and matrix continuity. Because the SEM images represent selected local fields of view, they were not used to quantitatively determine total porosity or pore-size distribution. The SEM analysis in this study was limited to morphological observation, and no site-specific elemental microanalysis was conducted.

## 3. Results and Discussion

### 3.1. Response Surface Results and Model Analysis

A Box–Behnken design (BBD) was adopted to develop the response surface experimental matrix for the mix proportions of FCB, resulting in 17 experimental runs. Among them, five center points were included to estimate the pure error and evaluate the stability of the model. The experimental design and corresponding response results are presented in Table 3. Noticeable variations in fluidity, initial setting time, final setting time, and 28 d uniaxial compressive strength were observed under different factor combinations, indicating that the replacement ratio, FA/CS ratio, and bentonite content all influence the workability and mechanical performance of FCB.

Second-order polynomial regression models were established using the least-squares method to describe the relationships between the response variables and the experimental factors. In Equations (1)–(4), Y_1_, Y_2_, Y_3_ and Y_4_ represent fluidity, initial setting time, final setting time, and 28 d uniaxial compressive strength, respectively. A, B, and C represent the coded values of the replacement ratio, FA/CS ratio, and bentonite content, respectively.

(1) $$Y_{1}=190.50-2.75A-1.44B-5.06C-5.00\mathrm{AB}+3.75\mathrm{AC}-0.38\mathrm{BC}-19{.31A}^{2}-8{.94B}^{2}-11{.94C}^{2}$$

(2) $$Y_{2}=92.40+5.38A+0.13B+7.75C+7.25\mathrm{AB}+3.00\mathrm{AC}\;-\;4.50\mathrm{BC}\;-\;0{.83A}^{2}-\;0{.33B}^{2}+1{.93C}^{2}$$

(3) $$Y_{3}=199.80+5.75A\;-\;0.38B+13.88C+3.25\mathrm{AB}+0.25\mathrm{AC}\;-\;4.50\mathrm{BC}\;-\;4{.65A}^{2}-\;4{.40B}^{2}-\;4{.40C}^{2}$$

(4) $$Y_{4}=3.814\;-\;1.514A+0.124B\;-\;0.250C+0.033\mathrm{AB}\;-\;0.195\mathrm{AC}+0.110\mathrm{BC}+0{.954A}^{2}+0{.094B}^{2}+0{.607C}^{2}$$

The full quadratic models were retained without stepwise reduction. The quadratic model form was specified in advance for the Box–Behnken design, and the lack-of-fit terms of all four models were not statistically significant. Although some individual terms were not statistically significant, they were retained to preserve model hierarchy and to maintain a consistent model structure for response surface analysis and multi-objective optimization. To avoid implying excessive numerical precision, the coefficients of the fluidity and setting-time models were rounded to two decimal places, whereas those of the compressive-strength model were rounded to three decimal places.

The significance analysis further shows that different response variables exhibit distinct sensitivities to the experimental factors. For the fluidity model, the quadratic terms A^2^ and C^2^ are significant, indicating that fluidity is mainly governed by the nonlinear effects of the replacement ratio and bentonite content. For the initial setting time, A, C, and the interaction term AB are significant, suggesting an interaction between the cement replacement level and the regulation of slurry structure. For the final setting time, C is identified as the dominant factor, indicating that bentonite content plays a controlling role in the later-stage setting process. For the 28 d uniaxial compressive strength, A, C, and the quadratic terms A^2^ and C^2^ are significant, with A showing the strongest effect. This indicates that the replacement ratio is the key factor governing the load-bearing capacity of the system. In contrast, the linear effect of B is relatively weak within the investigated range.

From the statistical parameters listed in Table 4, the R^2^ values for the models of fluidity, initial setting time, final setting time, and 28 d compressive strength are 0.8837, 0.8629, 0.8665, and 0.9849, respectively, with the strength model showing the best fitting performance. The coefficients of variation (C.V.) for all models are lower than 10%, and the Adeq Precision values are all greater than 4, indicating good repeatability and an adequate signal-to-noise ratio. It should be noted that the Pred R^2^ values for the fluidity and initial setting time models are relatively low, indicating limited predictive capability and suggesting that these models are more suitable for trend analysis within the experimental range. In contrast, the compressive-strength model exhibited the best statistical performance, with an R^2^ of 0.9849, an adjusted R^2^ of 0.9656, and a predicted R^2^ of 0.9356. The relatively close adjusted and predicted R^2^ values indicate good internal predictive consistency within the investigated design space. Therefore, the strength model was used as the primary basis for subsequent mix optimization. However, these statistical indicators do not establish predictive validity beyond the investigated factor ranges.

### 3.2. Response Surface Analysis

In order to further examine the effects of the replacement ratio (A), FA/CS ratio (B), and bentonite content (C) on the performance of FCB, response surface plots and contour maps were generated based on the developed quadratic regression models. The variation in each response was then interpreted in relation to changes in the governing factors.

#### 3.2.1. Fluidity

**Figure 4:**
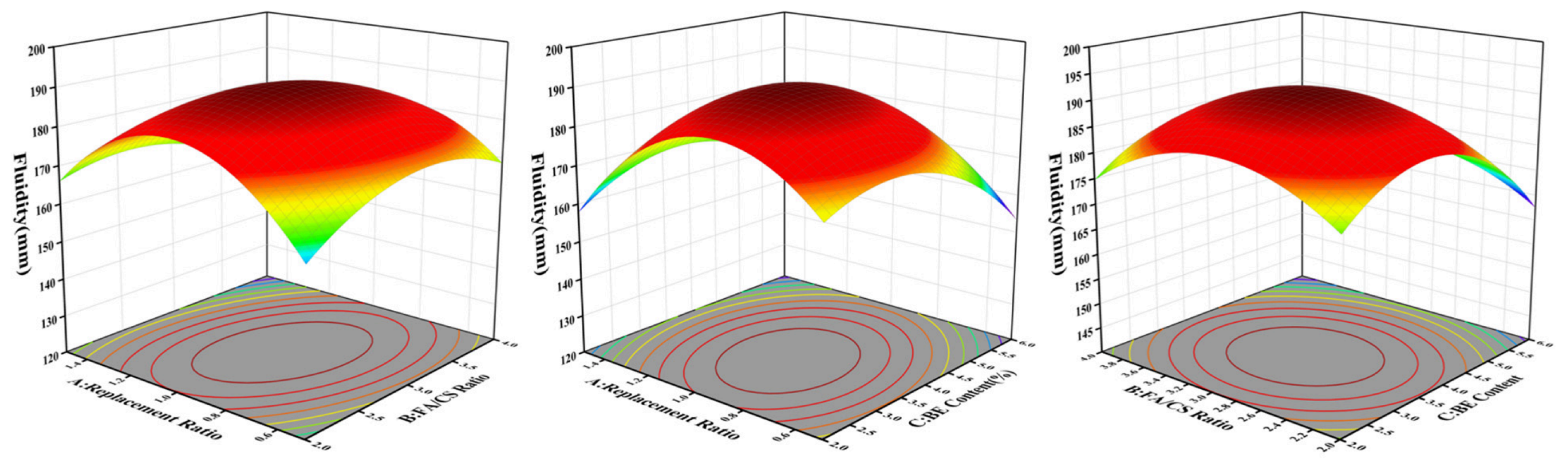
Response surface and contour plots of fluidity of FCB.

As depicted in Figure 4, the fluidity of FCB is mainly governed by the replacement ratio (A) and bentonite content (C), while the effect of the FA/CS ratio (B) is relatively limited. Fluidity exhibits a non-monotonic trend with respect to both A and C, increasing initially and then decreasing, which reflects a pronounced quadratic effect.

At low replacement ratios, the higher cement content leads to stronger interparticle bonding, which restricts slurry flow. As the replacement ratio increases to a moderate level, the spherical morphology of fly ash improves particle packing and promotes interparticle sliding, thereby enhancing flowability. However, further increases in A reduce the amount of effective cementitious components, weaken the structural stability of the slurry, and result in a decline in fluidity.

A similar nonlinear effect was observed for bentonite content. At a moderate dosage, the fine bentonite particles may improve particle gradation and slurry uniformity. At higher dosages, the layered fine particles and their affinity for water may reduce the amount of readily available mixing water and increase particle interactions, thereby lowering fluidity. Because the water absorption and specific surface area of the raw materials were not measured, this explanation should be regarded as a qualitative interpretation rather than a quantitatively verified mechanism. Consequently, higher fluidity was generally observed at intermediate levels of both A and C [29,30].

#### 3.2.2. Setting Time

**Figure 5:**
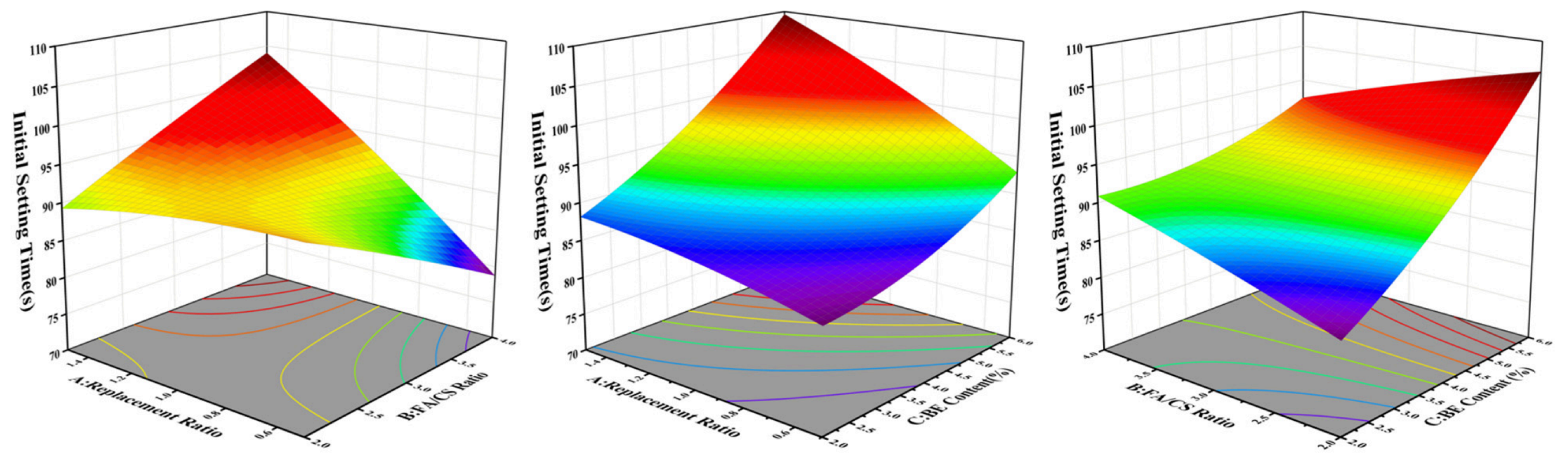

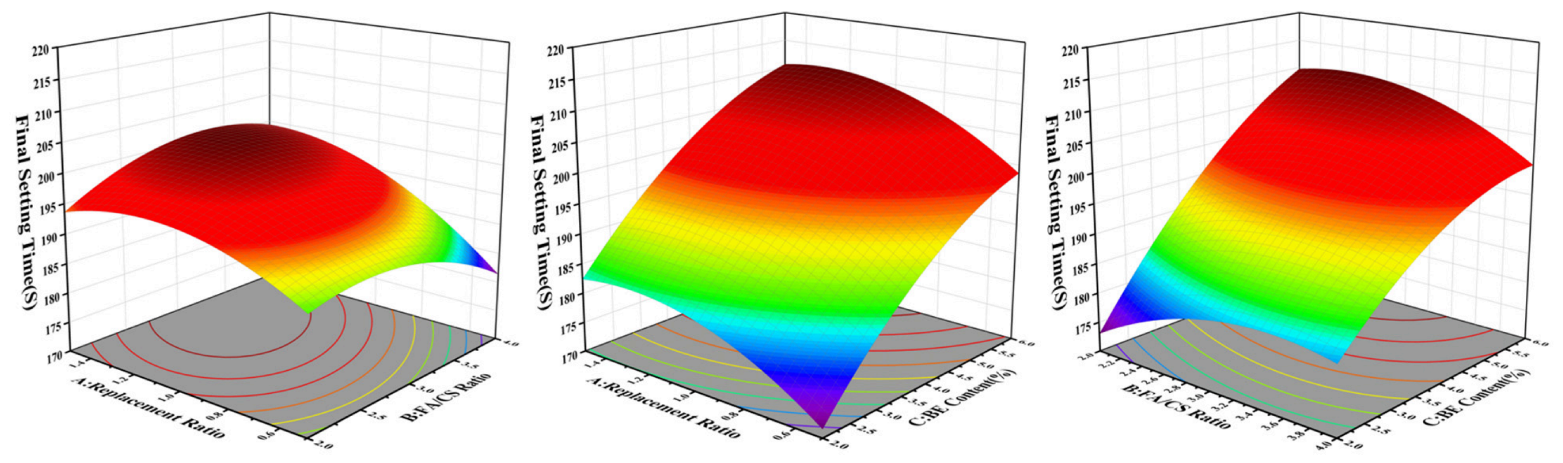
Response surface and contour plots of initial and final setting times of FCB.

Figure 5 shows that both the initial and final setting times are mainly controlled by the replacement ratio (A) and bentonite content (C), and both increase with increasing values of these factors, with C exerting a more pronounced influence.

An increase in A corresponds to a reduction in cement content, which slows down early hydration reactions and delays the setting process. In addition, the relatively low reactivity of fly ash means that its contribution to strength development relies more on later-stage reactions, providing limited structural support at early ages and further prolonging the setting time.

The influence of bentonite on setting behavior may be related to changes in water availability and particle interactions. With increasing bentonite content, its layered fine particles and affinity for water may reduce the amount of readily available mixing water and increase internal resistance within the slurry. These effects could delay cement hydration and the development of a continuous solid skeleton. However, because the water absorption and specific surface area of bentonite were not measured, the relative contributions of water uptake, dilution, and particle interactions cannot be quantified in the present study. Compared with the initial setting time, the final setting time was more sensitive to changes in bentonite content, indicating that bentonite exerted a more sustained influence on the later-stage setting process [29,31].

#### 3.2.3. 28 d Uniaxial Compressive Strength

**Figure 6:**
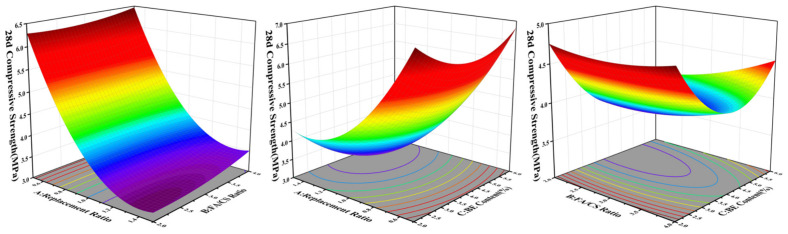
Response surface and contour plots of 28 d uniaxial compressive strength of FCB.

As shown in Figure 6, the 28 d uniaxial compressive strength of FCB is primarily governed by the replacement ratio (A), followed by the bentonite content (C), while the influence of the FA/CS ratio (B) is relatively minor.

With increasing A, the cement content decreases, leading to reduced formation of primary cementitious products such as C-(A)-S-H gel. This results in a less continuous matrix and a corresponding decrease in load-bearing capacity. Moreover, the activation of FA and the subsequent secondary reactions within the FA–CS fraction develop relatively slowly and cannot fully compensate, within 28 d, for the strength loss caused by the reduction in cement content. Consequently, the compressive strength decreases markedly with increasing replacement ratio [32].

The influence of bentonite content on strength also exhibited a nonlinear trend. A moderate dosage may improve particle distribution and contribute to a more uniform hardened matrix. In contrast, excessive bentonite may increase water demand, dilution, swelling tendency, or the formation of weak local interfaces, thereby reducing the load-bearing capacity. These possible mechanisms are inferred from the mechanical-property trends and SEM observations. Because water absorption, particle density, specific surface area, and quantitative pore-structure parameters were not measured, the individual contribution of each mechanism could not be independently verified in the present study [33].

Overall, higher strength values are generally obtained at low replacement ratios and moderate bentonite contents. This distribution is broadly consistent with the trends observed for fluidity and setting time.

To place the present results in the context of previously reported fly ash-containing backfill systems, the optimized FCB mixture achieved a measured fluidity of 173.5 mm and a 28 d uniaxial compressive strength of 5.96 MPa. Hu et al. [8] optimized an alkali-activated slag–fly ash cemented tailings backfill containing sodium silicate and NaOH and reported a fluidity of 280 mm and a 28 d compressive strength of 4.4 MPa under the optimized conditions. Compared with that alkali-activated system, the present FCB exhibited lower fluidity but higher 28 d strength under the respective optimized conditions. Nevertheless, this numerical comparison should be interpreted cautiously because the tailings properties, slurry concentration, binder-to-tailings ratio, activator composition, specimen dimensions, curing conditions, and testing procedures differed between the two studies.

Fly ash geopolymer backfill and alkali-activated slag–fly ash backfill generally rely on externally added alkaline activators to promote the dissolution and reaction of aluminosilicate precursors [9,10]. In contrast, the present system contains no externally added NaOH or sodium silicate. CS provides calcium and alkalinity, cement supplies conventional hydration products, and BE regulates water retention, particle packing, and slurry behavior. Unlike the pre-depolymerized FA–CS system reported by Zeng et al. [11] and the carbide slag-activated GGBS–FA system investigated by Li et al. [12], the present formulation requires neither pretreatment nor the incorporation of GGBS. Instead, FA, CS, and BE are directly incorporated into cemented whole-tailings backfill, and their proportions are optimized by simultaneously considering fluidity, setting time, and strength. This provides a relatively simple preparation route, although the strength of the system remains sensitive to increases in the cement replacement ratio.

### 3.3. Multi-Objective Optimization and Validation

To achieve a balance among workability, setting behavior, and mechanical performance of FCB, the numerical optimization module in Design-Expert 13 was employed for multi-objective optimization of the mix parameters. During the optimization process, fluidity, initial setting time, and final setting time were defined as range constraints, while the 28 d uniaxial compressive strength was set as the maximization objective. This configuration accounts for both the workability and controllability of the fresh slurry, as well as the load-bearing performance of the hardened backfill.

The optimization results indicated that the preferred region corresponded to a relatively low replacement ratio, a moderate FA/CS ratio, and a moderately high bentonite content. The recommended mix proportions were A = 0.58, B = 2.71, and C = 4.99%. This combination was located within the investigated factor ranges but was not included among the 17 runs used for model fitting. An independent confirmation experiment was therefore conducted at this point, and the results are presented in Table 5.

The measured fluidity, initial setting time, final setting time, and 28 d uniaxial compressive strength were 173.50 mm, 95 min, 200 min, and 5.96 MPa, respectively. The corresponding relative errors between the measured and predicted values were 1.80%, 2.49%, 0.80%, and 1.68%, all of which were below 5%. These results confirm the interpolation accuracy of the developed models at an untested point within the design space. No validation experiments were conducted outside the investigated ranges of A, B, and C. Therefore, the models should be applied only within the ranges considered in the Box–Behnken design, and extrapolation beyond these ranges is not recommended without additional experimental verification.

### 3.4. Microstructural Characteristics and Mechanism

#### 3.4.1. Phase Composition of Hydration Products

**Figure 7:**
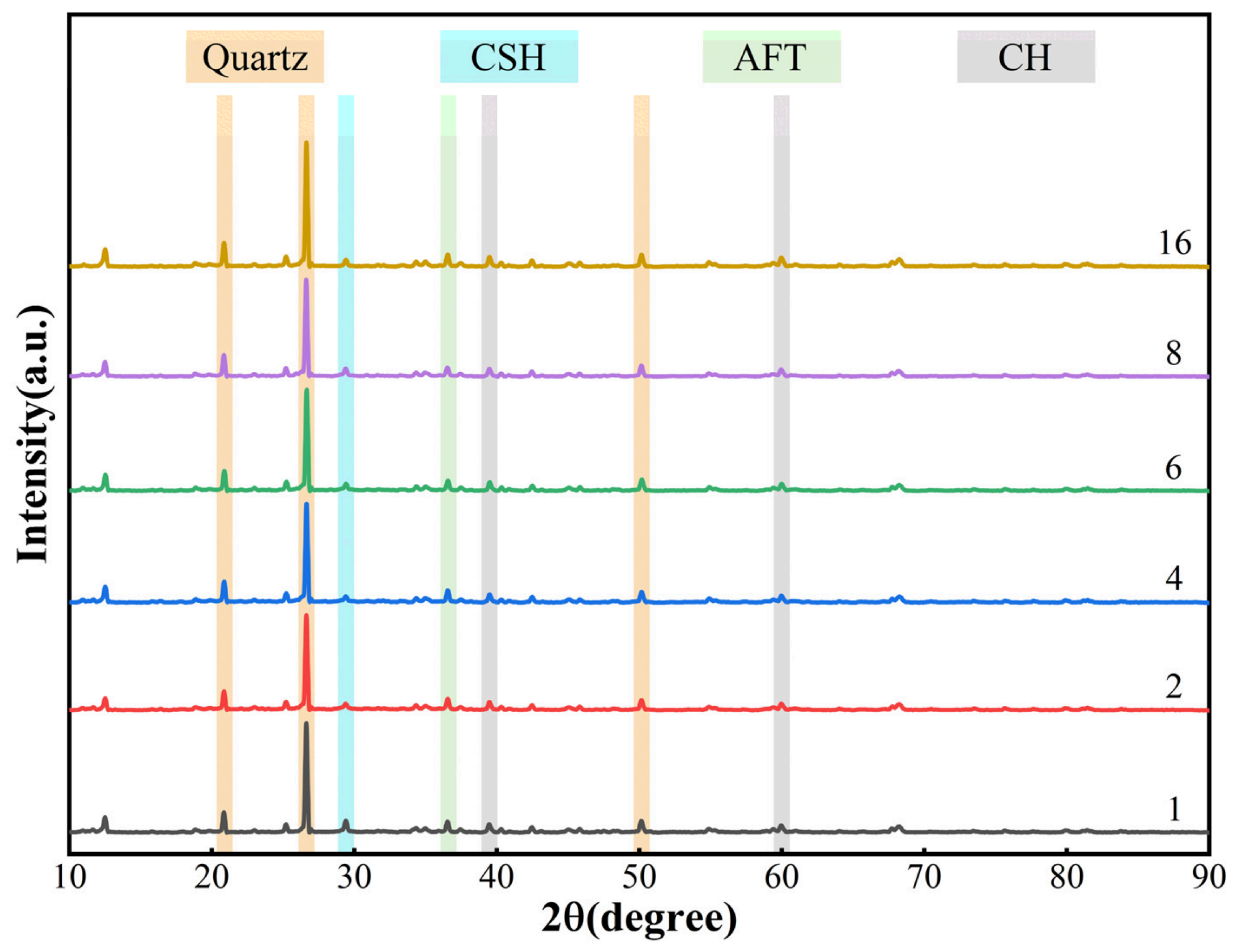
XRD patterns of hydration products of FCB.

XRD analysis was performed on representative specimens at 28 d, and the hydration products of FCB were identified accordingly, as shown in Figure 7. All specimens exhibit distinct quartz diffraction peaks, which mainly originate from the inert mineral components in the whole tailings. This indicates that the tailings primarily serve as a skeletal framework within the system.

In addition to quartz, broad diffuse features associated with poorly crystalline C-(A)-S-H gel and characteristic reflections of AFt were observed in all specimens, while a small amount of CH was detected in some mixtures [34]. No additional well-resolved crystalline phases were identified within the detection capability of the XRD analysis. However, the similarity of the major diffraction features does not necessarily indicate that the hydration products were identical in all mixtures. Variations in the replacement ratio, FA/CS ratio, and bentonite content may affect the relative amounts, degree of crystallinity, and chemical composition of the reaction products without producing clearly distinguishable new diffraction peaks.

At lower replacement ratios, the diffuse feature associated with C-(A)-S-H gel was more pronounced, indicating a greater amount of poorly crystalline cementitious products and a higher potential for forming a dense and continuous matrix. As the replacement ratio increased, the intensity of the gel-related feature and the CH reflections generally decreased, reflecting the reduction in cement content and the lower overall degree of hydration at 28 d [35]. Changes in the FA/CS ratio may also influence the balance between the aluminosilicate source and the calcium-rich alkaline environment, whereas bentonite may affect the reaction process through dilution, water retention, and its fine-particle filling effect.

No distinct diagnostic reflections attributable to hydrotalcite-like or zeolitic phases were observed in the present XRD patterns. Therefore, these phases were not identified in this study. Nevertheless, the possible presence of trace amounts or poorly crystalline forms cannot be completely excluded on the basis of conventional XRD analysis alone.

Overall, C-(A)-S-H gel and AFt were the dominant hydration products detectable by XRD, accompanied by a small amount of CH in some specimens. The investigated compositional variations mainly affected the relative amounts and structural development of these products. Therefore, the present interpretation is limited to the dominant XRD-detectable phases and does not exclude the possible formation of minor or poorly crystalline products.

#### 3.4.2. Microstructure and Morphological Characteristics

**Figure 8:**
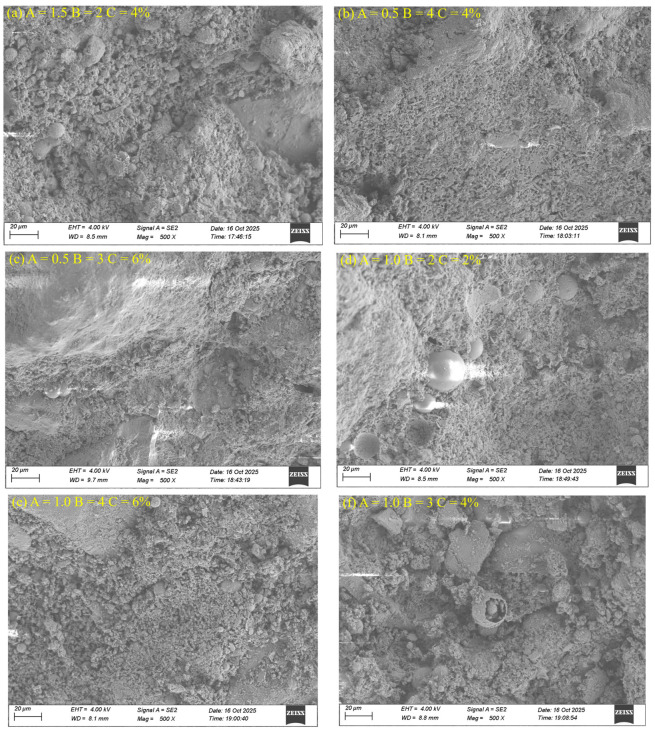
Low-magnification SEM images (500×) of FCB specimens under different mix proportions.

Six representative specimens were selected from the response surface design for SEM analysis. These specimens covered different regions of the design space and highlighted the effects of the main compositional factors. Figure 8a–f correspond to Samples 2, 3, 7, 9, 12, and 13, respectively.

The low-magnification SEM images show qualitative differences in the surface morphology of the selected specimens. In the examined fields of view, specimens with higher replacement ratios appeared locally looser and exhibited more visible voids and discontinuous particle-contact regions. In contrast, specimens with lower replacement ratios generally showed more continuous particle contacts and fewer visible voids. At moderate bentonite contents, the particle distribution appeared relatively uniform, whereas higher bentonite contents were associated with more visible voids and local interfacial gaps. These observations are limited to the selected SEM fields and should not be interpreted as quantitative evidence of changes in total porosity or pore-size distribution.

**Figure 9:**
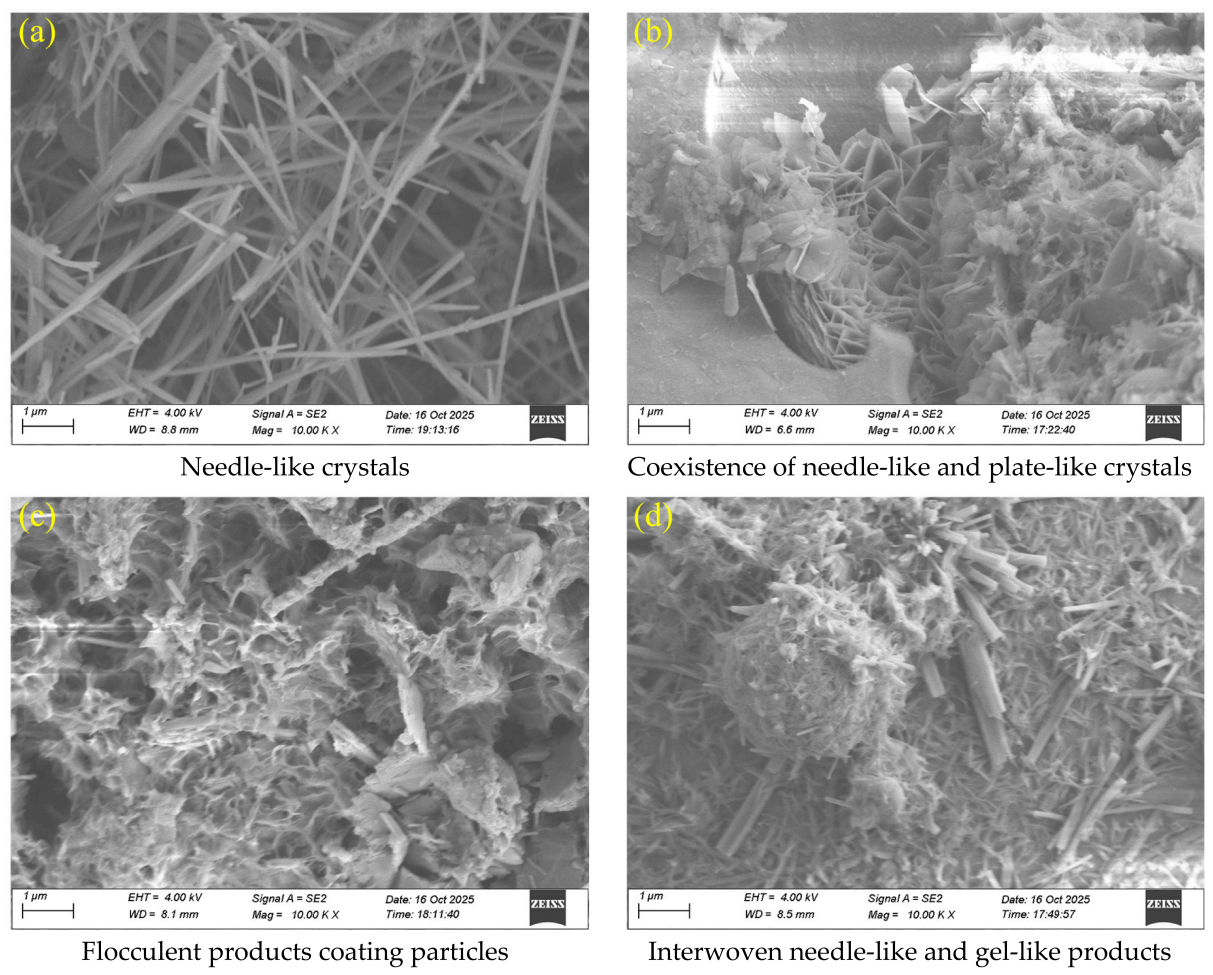
High-magnification SEM images (10,000×) showing typical morphologies observed in FCB: (**a**) needle-like crystals tentatively associated with AFt; (**b**) coexistence of needle-like and plate-like crystals; (**c**) flocculent products tentatively associated with C-(A)-S-H gel coating the particles; and (**d**) interwoven needle-like and gel-like products forming an apparently interconnected morphology.

The high-magnification SEM images in Figure 9 show several characteristic morphologies, including needle-like crystals, flocculent or network-like products coating the particles, and occasional plate-like crystals. Based on their morphological characteristics and the phases identified by XRD, these features were tentatively associated with AFt, C-(A)-S-H gel, and CH, respectively [36,37]. In some observed regions, needle-like and gel-like products occurred around and between the particles and formed an apparently interconnected morphology. Such distributions may contribute to particle bridging and matrix continuity. However, SEM and XRD alone cannot quantify the abundance of these products, their contribution to interparticle bonding, or the extent of pore filling.

Taken together, the XRD and SEM results indicate an association between the observed reaction-product morphologies, the apparent continuity of the selected microstructural regions, and the measured compressive-strength trends. Lower replacement ratios and appropriate bentonite contents were generally associated with more continuous morphologies in the examined SEM fields. These observations provide qualitative support for the macroscopic results but do not constitute quantitative evidence of pore refinement or bulk-matrix densification. Additional techniques, such as mercury intrusion porosimetry or X-ray computed tomography, would be required to confirm changes in total porosity and pore-size distribution.

Because site-specific EDX analysis was not performed, the assignment of the observed morphologies to individual hydration products should also be regarded as qualitative and is supported primarily by the XRD results and previously reported morphological characteristics.

### 3.5. Preliminary Economic and Environmental Considerations

The optimized FCB mixture partially replaces cement with FA and CS while utilizing whole tailings as the backfill aggregate. Because cement is generally one of the major contributors to the material cost and embodied carbon of cemented mine backfill, its partial replacement provides potential economic and environmental benefits [5,6,7]. In addition, FA and CS are incorporated directly without chemical pretreatment or the addition of external alkaline activators such as NaOH and sodium silicate. This relatively simple preparation route may reduce additional chemical consumption, handling requirements, and processing costs. The use of whole tailings, FA, and CS also promotes the cooperative utilization of industrial solid wastes and may reduce the demand for surface waste disposal.

Nevertheless, the optimized mixture was selected primarily by balancing fluidity, setting behavior, and 28 d compressive strength rather than by directly minimizing cost or carbon emissions. Although the optimized mixture, with A = 0.58, B = 2.71, and C = 4.99%, achieved a measured 28 d compressive strength of 5.96 MPa, the present experimental data are insufficient to quantify its actual economic savings or environmental benefits. The practical performance may be affected by local material prices, transportation distances, moisture conditioning, storage, mixing and pumping energy, and the additional consumption of bentonite and superplasticizer.

Therefore, the economic and environmental advantages discussed in this study should be regarded as preliminary. A site-specific techno-economic analysis and life-cycle assessment are required before large-scale engineering application. Such an assessment should consider the production and transportation of all constituent materials, energy consumption during backfill preparation, avoided solid-waste disposal, field pumping requirements, and the long-term environmental stability of the backfill. A functional unit such as 1 m^3^ of backfill satisfying specified workability and strength requirements would allow meaningful comparison with conventional cement-based backfill.

## 4. Conclusions

Fly ash–carbide slag–bentonite-based whole-tailings backfill (FCB) was investigated using a Box–Behnken response surface design. The effects of the replacement ratio (A), FA/CS ratio (B), and bentonite content (C) on fluidity, setting time, and 28 d uniaxial compressive strength were evaluated. The underlying mechanisms were examined using XRD and SEM. The main conclusions are as follows:

(1) Quadratic response surface models for fluidity, initial setting time, final setting time, and 28 d uniaxial compressive strength passed significance tests, and the lack-of-fit terms were not significant. The models adequately describe the relationships between factors and responses within the studied range. The compressive strength model shows the best fit and predictive capability and can be used as the primary basis for mix optimization. The models for fluidity and initial setting time show limited predictive capability and are more suitable for trend analysis within the experimental range.
(2) The sensitivities of the response variables to the factors differ. The replacement ratio and bentonite content are the main factors affecting FCB performance, while the FA/CS ratio plays a secondary role within the investigated design space. Fluidity is governed by the quadratic effects of the replacement ratio and bentonite content. Both initial and final setting times increase with increasing replacement ratio and bentonite content. The 28 d uniaxial compressive strength is mainly controlled by the replacement ratio and decreases as the replacement ratio increases.
(3) Multi-objective optimization based on a desirability function indicated that the preferred region corresponded to a relatively low replacement ratio, a moderate FA/CS ratio, and a moderately high bentonite content. The optimal mix proportions were A = 0.58, B = 2.71, and C = 4.99%. The independent confirmation experiment conducted at an untested point within the design space agreed well with the model predictions, with relative errors below 5% for all responses. This result supports the applicability of the models for interpolation and mix optimization within the investigated factor ranges; their use beyond these ranges requires additional validation.
(4) XRD indicated that the dominant detectable hydration products of FCB were C-(A)-S-H gel and AFt, accompanied by a small amount of CH in some specimens. In the selected SEM fields, lower replacement ratios and appropriate bentonite contents were generally associated with more continuous particle contacts and fewer visible voids, whereas excessive bentonite contents were associated with more apparent voids and interfacial gaps. These observations are qualitative and do not provide quantitative evidence of pore refinement or bulk-matrix densification. Because site-specific EDX and quantitative pore-structure analyses were not performed, the assignment of individual morphologies and the interpretation of pore-related mechanisms remain tentative.
(5) The optimized FCB mixture has potential economic and environmental advantages because it partially replaces cement and enables the cooperative utilization of FA, CS, and whole tailings without additional alkaline activators or chemical pretreatment. However, these advantages have not yet been quantified, and site-specific cost analysis and life-cycle assessment are required before large-scale application.

## Figures and Tables

**Table 1:** Main chemical compositions of the raw materials (wt.%).

Materials	SiO_2_	Al_2_O_3_	Fe_2_O_3_	CaO	MgO	SO_3_	Na_2_O	K_2_O	Others
CE	23.49	7.82	2.52	58.05	1.83	3.10	1.23	1.13	0.82
FA	51.39	36.83	2.81	3.03	0.58	1.37	0.46	1.71	1.81
CS	3.62	1.92	0.17	93.08	0.23	0.41	0.22	-	0.34
BE	52.16	21.50	4.46	15.67	2.16	0.10	0.23	2.51	-
TA	71.69	12.65	8.89	0.99	2.88	0.42	0.19	1.28	-

Note: “-” indicates not detected or not available.

**Table 2:** Factor levels and codes used in the response surface design.

Factor	−1	0	1
A	0.5	1.0	1.5
B	2	3	4
C	2	4	6

**Table 3:** Experimental design and results of the response surface analysis.

Sample	Factor A	Factor B	Factor C (%)	Fluidity (mm)	Initial Setting Time (min)	Final Setting Time (min)	28 d Compressive Strength (MPa)
1	0.5	2	4	162.5	91	187	6.35
2	1.5	2	4	172	86	192	3.13
3	0.5	4	4	162.5	82	183	6.53
4	1.5	4	4	152	106	201	3.44
5	0.5	3	2	178	84	171	6.95
6	1.5	3	2	160	90	182	4.44
7	0.5	3	6	151	91	199	6.7
8	1.5	3	6	148	109	211	3.41
9	1	2	2	167.5	83	175	4.68
10	1	4	2	172.5	87	180	4.71
11	1	2	6	167.5	110	211	4.1
12	1	4	6	171	96	198	4.57
13	1	3	4	183	86	210	3.89
14	1	3	4	196	95	200	4.03
15	1	3	4	189.5	90	189	3.46
16	1	3	4	191	92	193	3.59
17	1	3	4	193	99	207	4.1

**Table 4:** Analysis of variance (ANOVA) results of the response surface regression models.

Source of Variance	DF	Fluidity (mm)			Initial Setting Time (min)			Final Setting Time (min)			28 d Compressive Strength (MPa)		
		Mean Square	F Value	*p* Value	Mean Square	F Value	*p* Value	Mean Square	F Value	*p* Value	Mean Square	F Value	*p* Value
Model	9	355.59	5.91	0.0144	117.46	4.9	0.024	245.91	5.05	0.0221	2.77	50.88	<0.0001
A	1	60.5	1.01	0.3494	231.13	9.63	0.0172	264.5	5.43	0.0526	18.33	336.41	<0.0001
B	1	16.53	0.2747	0.6164	0.125	0.0052	0.9445	1.13	0.0231	0.8835	0.1225	2.25	0.1774
C	1	205.03	3.41	0.1074	480.5	20.03	0.0029	1540.12	31.61	0.0008	0.5	9.18	0.0191
AB	1	100	1.66	0.2384	210.25	8.76	0.0211	42.25	0.8672	0.3827	0.0042	0.0775	0.7887
AC	1	56.25	0.9346	0.3659	36	1.5	0.2602	0.25	0.0051	0.9449	0.1521	2.79	0.1387
BC	1	0.5625	0.0093	0.9257	81	3.38	0.1088	81	1.66	0.2382	0.0484	0.8882	0.3773
A^2^	1	1570.41	26.09	0.0014	2.87	0.1194	0.7398	91.04	1.87	0.2139	3.83	70.36	<0.0001
B^2^	1	336.33	5.59	0.0501	0.4447	0.0185	0.8955	81.52	1.67	0.2369	0.0374	0.6864	0.4347
C^2^	1	600.02	9.97	0.016	15.6	0.6503	0.4465	81.52	1.67	0.2369	1.55	28.45	0.0011
Residual	7	60.19	-	-	23.99	-	-	48.72	-	-	0.0545	-	-
Lack of fit	3	109.1	4.64	0.0861	23.58	0.9705	0.4892	7.42	0.0931	0.96	0.0239	0.3088	0.8192
Pure error	4	23.5	-	-	24.3	-	-	79.7	-	-	0.0774	-	-
R^2^		0.8837			0.8629			0.8665			0.9849		
Adj R^2^		0.7341			0.6866			0.6948			0.9656		
Pred R^2^		−0.4866			−0.048			0.6656			0.9356		
C.V./%		4.52			5.28			3.61			5.08		
Adeq Precision		6.2918			8.2185			7.3317			20.9524		

Note: “-” indicates not detected or not available.

**Table 5:** Multi-objective optimization results and validation of FCB.

Item	A	B	C (%)	Fluidity (mm)	Initial Setting Time (min)	Final Setting Time (min)	28 d Compressive Strength (MPa)
Predicted	0.58	2.71	4.99	170.38	92.63	198.4	5.86
Measured	-	-	-	173.50	95	200	5.96
Error (%)	-	-	-	1.80	2.49	0.80	1.68

Note: “-” indicates not detected or not available.

## Data Availability

The original contributions presented in this study are included in the article. Further inquiries can be directed to the corresponding author.

## References

[1] Wu A., Wang Y., Ruan Z., Xiao B., Wang J., Wang L. (2024). Key theory and technology of cemented paste backfill for green mining of metal mines. Green Smart Min. Eng..

[2] Xu W., Zhang Y., Zuo X., Hong M. (2020). Time-dependent rheological and mechanical properties of silica fume modified cemented tailings backfill in low temperature environment. Cem. Concr. Compos..

[3] Jiao H., Zhang W., Yang Y., Chen X., Yang L., Shen H., Rong Y., Zhang H. (2023). Static mechanical characteristics and meso-damage evolution characteristics of layered backfill under the condition of inclined interface. Constr. Build. Mater..

[4] Belibi Tana A.E., Yin S., Wang L. (2021). Investigation on mechanical characteristics and microstructure of cemented whole tailings backfill. Minerals.

[5] Fang K., Zhang J., Cui L., Haruna S., Li M. (2023). Cost optimization of cemented paste backfill: State-of-the-art review and future perspectives. Miner. Eng..

[6] Cao H., Gao Q., Zhang X., Guo B. (2022). Research progress and development direction of filling cementing materials for filling mining in iron mines of China. Gels.

[7] Liu W., Zhou Z., Li H., Zhang T., Mai Q., Li C. (2025). Research on the performance of low carbon mine filling cementing material based on red mud: A comprehensive review. Case Stud. Constr. Mater..

[8] Hu J., Meng Z., Gao T., Dong S., Ni P., Li Z., Yang W., Wang K. (2024). Optimization of proportions of alkali-activated slag-fly ash-based cemented tailings backfill and its strength characteristics and microstructure under combined action of dry-wet and freeze-thaw cycles. Materials.

[9] Sun Q., Tian S., Sun Q., Li B., Cai C., Xia Y., Wei X., Mu Q. (2019). Preparation and microstructure of fly ash geopolymer paste backfill material. J. Clean. Prod..

[10] Liu S., Wang Y., Wu A., Shi D., Yang S., Ruan Z., Song X., Zhang M. (2024). Early mechanical strength, hydration mechanism and leaching behavior of alkali-activated slag/fly ash paste filling materials. J. Build. Eng..

[11] Zeng J., Liu Q., Yang J., He X., Su Y., Zhang Q., Strnadel B. (2024). Recycling of fly ash and carbide slag in low-carbon cement composites by pre-depolymerization and pre-dissolution treatment. J. Build. Eng..

[12] Li H., Wang R., Wei M., Lei N., Wei T., Liu F. (2024). Characteristics of carbide-slag-activated GGBS-fly ash materials: Strength, hydration mechanism, microstructure, and sustainability. Constr. Build. Mater..

[13] Hasan K., Putra Jaya R., Yahaya F.M. (2024). Application of bentonite in cement-based composites: A review of current status, challenges and future prospects. J. Build. Eng..

[14] Fode T.A., Jande Y.A.C., Kivevele T. (2024). Effects of raw and different calcined bentonite on durability and mechanical properties of cement composite material. Case Stud. Constr. Mater..

[15] Myers R.H., Montgomery D.C., Anderson-Cook C.M. (2016). Response Surface Methodology: Process and Product Optimization Using Designed Experiments.

[16] Huang M., Chen L., Zhang M., Zhan S. (2022). Multi-objective function optimization of cemented neutralization slag backfill strength based on RSM-BBD. Materials.

[17] Sun K., Fall M. (2023). Response surface methodology-based characterization and optimization of fibre reinforced cemented tailings backfill with slag. Int. J. Min. Reclam. Environ..

[18] Zhao Y., Zhou X., Zhou Q., Zhu H., Cheng F., Chen H. (2024). Optimization design of anhydrous phosphogypsum-based backfill materials incorporating carbide slag and blast furnace slag by response surface method. Miner. Eng..

[19] Standardization Administration of the People’s Republic of China (2017). Fly Ash Used for Cement and Concrete.

[20] Snellings R., Suraneni P., Skibsted J. (2023). Future and emerging supplementary cementitious materials. Cem. Concr. Res..

[21] Duan K., Wang J., Liu Z., Li X., Zhang J., Wang X., Wang D. (2025). Flowability and in-situ phase evolution of Na_2_CO_3_-carbide slag-activated blast furnace slag and fly ash. Constr. Build. Mater..

[22] Jiang H., Ke G., Li Z. (2024). Synergistic activation mechanism and long-term properties of a solid waste cementitious material based on red mud and calcium carbide slag. Constr. Build. Mater..

[23] Zhao X., Lu J.-X., Tian W., Li S., Qi X., Shui Z., Poon C.S. (2023). Natural bentonite as an internal curing agent in the production of eco-friendly ultra-high performance concrete with low autogenous shrinkage. J. Clean. Prod..

[24] Box G.E.P., Behnken D.W. (1960). Some new three level designs for the study of quantitative variables. Technometrics.

[25] Ferreira S.L.C., Bruns R.E., Ferreira H.S., Matos G.D., David J.M., Brandão G.C., da Silva E.G.P., Portugal L.A., dos Reis P.S., Souza A.S. (2007). Box-Behnken design: An alternative for the optimization of analytical methods. Anal. Chim. Acta.

[26] (2005). Test Method for Fluidity of Cement Mortar.

[27] (2011). Test Methods for Water Requirement of Normal Consistency, Setting Time and Soundness of the Portland Cement.

[28] (2019). Standard for Test Methods of Concrete Physical and Mechanical Properties.

[29] Sayed D.G., Ramadan M., Mohsen A. (2025). Valorizing calcinated-bentonite in the production of hydrothermally cured geopolymers with high mechanical-efficacy. Process Saf. Environ. Prot..

[30] Wu Y., Li F., Yang J. (2025). Synthesis of low-alkaline based all-solid-waste cemented tailings backfill: Design methodology, macro-micro properties, and benefit evaluation. Chem. Eng. J..

[31] Cai J., Sun C., Zhang J., Cheng X., Tang N. (2025). Mix design optimization and performance prediction of multi-source solid waste-based backfill materials using response surface methodology and BP neural network. Results Eng..

[32] Zheng Y., Liu C., Wang J., Pan H., Yan X., Wagland S.T., Zhang G., Cheng L. (2025). High-dosage carbide slag-based cementitious material: Workability, mechanical strength, and carbonation behavior with the performance enhancement. Case Stud. Constr. Mater..

[33] Li Y., Wang S., Fei K. (2025). Study on the effect of bentonite hydration on engineering and mechanical properties of plastic concrete antifouling barriers in landfills. Process Saf. Environ. Prot..

[34] Yang G., Li C., Xie W., Yue Y., Kong C., Li X. (2024). Effect of carbide slag and steel slag as alkali activators on the key properties of carbide slag-steel slag-slag-phosphogypsum composite cementitious materials. Front. Mater..

[35] Xu W., Cao P., Tian M. (2018). Strength development and microstructure evolution of cemented tailings backfill containing different binder types and contents. Minerals.

[36] Gan D., Li H., Liu Z. (2025). Experimental study on dynamic characteristics of cemented tailings backfill under different tailings gradation. Appl. Sci..

[37] Huang J., Deng L., Gao H., Wu C., Li J., Zhu D. (2025). Enhancing tensile performance of cemented tailings backfill through 3D-printed polymer lattices: Mechanical properties and microstructural investigation. Materials.

